# Occurrence, environmental implications and risk assessment of Bisphenol A in association with colloidal particles in an urban tropical river in Malaysia

**DOI:** 10.1038/s41598-020-77454-8

**Published:** 2020-11-23

**Authors:** Zakariya Nafi’ Shehab, Nor Rohaizah Jamil, Ahmad Zaharin Aris

**Affiliations:** 1 Department of Environment, Faculty of Forestry and Environment, Universiti Putra Malaysia, UPM, 43400 Serdang, Selangor Malaysia; 2 International Institute of Aquaculture and Aquatic Sciences (i-AQUAS), Lot 960 Jln Kemang 6, 71050 Port Dickson, Negeri Sembilan Malaysia

**Keywords:** Environmental sciences, Hydrology, Endocrinology, Risk factors, Chemistry

## Abstract

Phase distribution of emerging organic contaminants is highly influential in their presence, fate and transport in surface water. Therefore, it is crucial to determine their state, partitioning behaviour and tendencies in water environments. In this study, Bisphenol A was investigated in both colloidal and soluble phases in water. BPA concentrations ranged between 1.13 and 5.52 ng L^−1^ in the soluble phase and n.d-2.06 ng L^−1^ in the colloidal phase, respectively. BPA was dominant in the soluble phase, however, the colloidal contribution ranged between 0 and 24% which implied that colloids can play a significant role in controlling BPA’s transportation in water. Urban and industrial areas were the main sources of BPA while forest areas displayed lower levels outside the populated domains. pH levels were between 6.3 and 7.4 which might have affected BPA’s solubility in water to some extent. The particle size distribution showed that the majority of the particles in river samples were smaller than 1.8 µm in diameter with a small presence of nanoparticles. Zeta potential varied between − 25 and − 18 mV, and these negative values suggested instability of particles. Furthermore, BPA was positively correlated with BOD, COD and NH_3_–N which might indicate that these organic compounds were released concurrently with BPA. RQ assessment showed low levels of risk towards algae and fish in the study area.

## Introduction

Bisphenol A (BPA) is a semi-volatile organic substance widely utilized in industrial production and found in daily used products. BPA, a phenolic xenoestrogen, is a monomer applied in epoxy resins’ production, polycarbonate and as an additive in plastic material e.g. water pipes and polyvinyl chloride^1^. Hence, the global market for BPA is forecast to reach 7.3 million metric tons by 2020^2^. This compound is often detected in wastewater effluent from industrial and urban sources at concentrations varying between 0.23 and 149 µg/L^3^. Studies in Spain and Greece have revealed that only around 68–87% of BPA is removed by conventional processes in sewage treatment plants^4^, whereas the rest ends up in the receiving surface and coastal waters^5^. Furthermore, biomonitoring studies indicate an internal exposure risk to BPA among human beings all over^6^, even though this chemical is biotransformed quickly and excreted in urine. Although BPA has a low affinity for estrogen receptors in comparison with other compounds (e.g. 17β-estradiol), it still causes parallel alternations in certain cell functions. Moreover, BPA in nanogram ranges was found to be effective at preventing the release of a vital adipokine which shields humans from metabolic syndrome^7^. Cardiovascular disease and diabetes in humans may also be associated with higher exposure to BPA compounds^8^.

Several organic compounds, such as BPA, are hydrophobic by definition, and therefore it is frequently presumed that these compounds can be removed from the water phase by the removal of particles^9^. Moreover, on top of the soluble and particulate phase, organic contaminants may attach to colloids as well, sometimes referred to as “the third phase”. Studies have illustrated that organic compounds can get adsorbed to organic colloids in soil, groundwater and surface water. Pollutants associated with colloidal particles can undergo different transformation processes which can be stimulated by sunlight and the presence of oxides or other compounds^9^.

Most studies investigate occurrence and distribution of pollutants in water with a specific focus on its “dissolved phase”, which though arbitrarily, but is conveniently defined as any substances that pass through a membrane filter with pores between 0.22 and 0.70 µm^10^. However, it has become widely acknowledged that small colloidal particles, which are abundant in the water systems, actually pass through these membranes and thus are frequently overlooked for their role in pollutants’ behaviour and distribution. Therefore, this traditional dissolved phase can be separated further into an extra filtrate termed as the “soluble phase” as well as a spectrum of colloidal particles^11^. Prior studies proposed that colloidal particles can be a significant sink or milieu for certain pharmaceuticals^12^. Lately, they have attracted more consideration due to their high pollutant reactivity and mobility implications.

Colloids are particles comprised of broad range of atomic structures and chemical compositions, and they have different physicochemical characteristics and origins. Nonetheless, the explicit interaction between Endocrine Disrupting Chemicals (EDCs) and colloids are still largely unknown^13^. Specifically, the main parameters in the interaction between colloids and EDCs are still unidentified, and also how these parameters are influenced by the different environmental factors, this has not been addressed appropriately. Since colloidal bound pollutants can exist with different sizes, charges and stoichiometry in comparison to soluble substances, they can travel farther distances in water. Thus, it is highly important to investigate and study the partitioning of organic pollutants among the dissolved phase, the soluble phase and the colloidal phase^9^.

In Malaysia, rivers supply about 98% of the country's potable water. Most of these rivers receive industrial and urban wastewater or treated effluent mainly processed by traditional water treatment practices^14^. However, several reports have stated that numerous organic pollutants such as BPA and pharmaceuticals aren’t removed completely throughout the treatment process^15,16^. Furthermore, rivers in Malaysia are extremely turbid; Deforestation and changing land uses, particularly the conversion of forests into oil palm plantations, have adversely impacted the water quality in rivers and the high volume of total suspended solids (TSS) have caused extra turbidity related issues in rivers. As a result of that, colloidal particles tend to form a suitable medium for pollutants’ adsorption due to their high surface area compared to other natural components in water, this association can take place through different mechanisms such as ion exchange or surface interactions. Furthermore, colloidal particles have the ability to carry pollutants to long distances and increase their distribution in the aquatic system. The major risk lies in the newly established Pahang-Selangor Raw Water Transfer (PSRWT) project, with its intake (Semantan water intake) located downstream of Bentong River. This project is one of the biggest projects in South-East Asia and is a vital long-term raw water supply to the population in Klang Valley. Therefore, Knowledge on phase distribution of organic pollutants such as BPA in urban waters will aid in estimating organic pollutants’ mobility, toxicity and associated risks on this water intake.

The aim of this work is to illustrate the phase distribution of Bisphenol A in Bentong River in Pahang State, Malaysia. The objectives are (1) determine the concentration of BPA in the colloidal and soluble phases; (2) characterize the colloidal particles in surface water; (3) investigate the potential role of water quality parameters in BPA distribution in different phases. Such knowledge will be vital to assess the multiphase fate and transport as well as the potential risks of BPA compounds in such an important drinking water resource.

## Materials and methods

### Sampling

**Figure 1:**
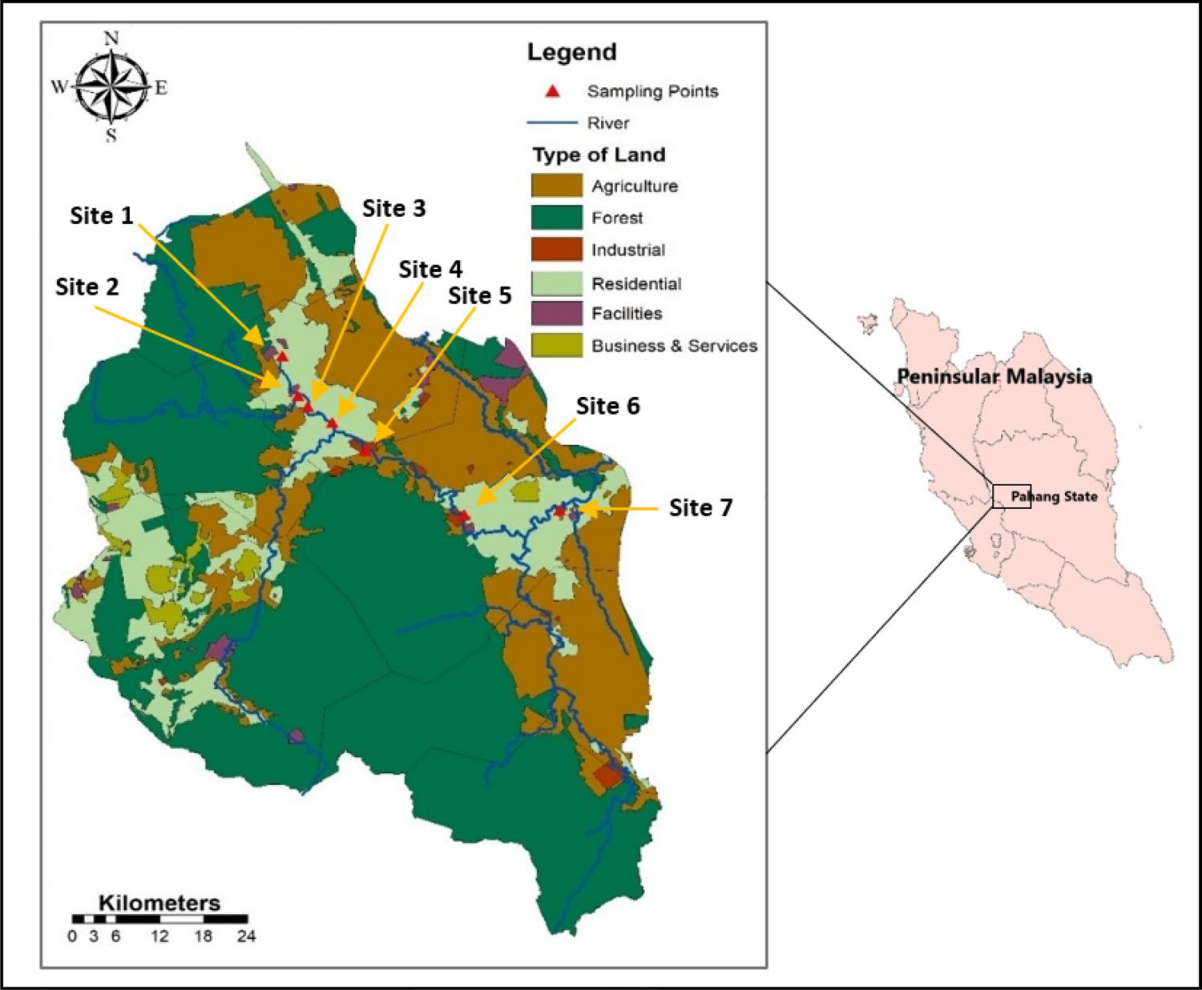
Location map and sampling sites of study area in Bentong, Pahang State, Malaysia.

Water samples were collected in two field trips along Bentong River during the normal and wet seasons (March and November, 2019). Systematic sampling, occasionally called interval sampling, which involves setting a gap or interval between each sampling site, has been adopted in this particular study. This strategy, whereby sites typically have an equal interval distance between them, is preferred since it minimizes bias in site selection and illustrates any changes in river conditions. Sampling sites were selected based on the current location of sewage effluent discharge points along Bentong River and its main tributaries. Furthermore, sampling sites located upstream and downstream were included to fully examine the river water quality in the study area. There were a set of 7 sampling stations that have been marked for the primary data collection and field observation (Fig. 1), the last two sites (sites 6 and 7) were outside municipality borders of Bentong District. Multiple STPs were also located along this river reach and with the accumulation of the previous treatment plants, these two sites were likely to bear the effects of the pollution load inside the district. Moreover, these last two sites were selected purposely to examine the water quality before abstraction by the Semantan water intake. A comprehensive schematic diagram of Bentong River, sampling locations and existing STPs (Fig. S1) can be found in the supplementary.

Water analysis and sampling practices were consistently carried out using grab sampling method where samples were taken at a single depth of about one meter below the water surface. All in-situ water parameters were recorded at site using a portable analytical probe (YSI Multiparameter Probe 556 and YSI Multiparamerer Professional series). Regarding the parameters that needed ex-situ analysis in laboratory facilities, samples were transported using specified Amber glass lab bottles to the laboratory in a cooler with dry icepacks and preserved according to APHA procedures. All analysis was done in commercial laboratory facilities. Analysis methods used for all water quality parameters were in accordance with the methods established by APHA in Standard Method Analysis of Water and Waste Water (APHA 2010). Regarding BPA analysis, duplicate samples (500 mL) were collected from the seven sampling sites mentioned above along Bentong River in November 2019. These duplicates were used to determine the precision of the sampling technique.

### Map generation

Digital elevation model (12.5 resolution) maps and data interpreted from 2011 Alaska Satellite Facility (ASFs) (Vertex) images were used to delineate and extract the stream network within Bentong watershed. Satellite images were geometrically corrected using ERDAS IMAGINE software (2016 version), afterwards the images were geo-referenced and subdivided according to an Area of Interest (AOI) concept. Images were then analysed by ascribing spectral signatures per pixel and distinguishing the study area into six categories according to certain digital number values for the discrete landscape components. These six categories were specified as residential area, forest, agriculture, industrial area, facilities and businesses and services. Each type was assigned a distinctive identity with a certain colour for proper distinction. Training samples were designated for each land cover type by delimiting polygons around representative spots. Spectral signatures for the specific land covers were registered using the pixels surrounded by these polygons. Finally, a maximum likelihood algorithm was applied to classify the satellite images, this classification relies on the representative pixels of the desired categories. Land use and cover composition map (Fig. 1) was produced using ArcGIS 10.2 (ESRI, Malaysia).

### Colloidal isolation

After transporting the samples to the laboratory, water samples were filtered via 1 µm glass fibre filters and divided into a particulate fraction and a traditionally dissolved phase. This conventionally dissolved phase was subdivided further into a colloidal phase and a soluble phase via cross-flow ultrafiltration (CFUF)^17^. The filtered water samples were separated via a 1 kDa ultrafiltration membrane into retentates (1 kDa–1 µm) which contained the colloids, and permeates (< 1 kDa) which represented the soluble phase. The retentate was redirected back to the feed container which made it more concentrated with time. Initially, the ultrafiltration system was cleaned by re-circulating 0.1 M NaOH solution for 1 h and then with feedwater. At the end of isolation, the colloidal phase and soluble phase for BPA were set for extraction.

The concentration of BPA in colloidal phase (C_c_) can be assessed from its level in the retentate (C_r_) and permeate (C_p_) and the concentration factor (cf) as in Eq. (1):

1 $$ {\text{C}}_{{\text{c}}} = {\text{C}}_{{\text{r}}} - {\text{C}}_{{\text{p}}} {\text{/cf}} $$

While the concentration factor can be calculated from the volumes of retentate (V_r_) and permeate (V_p_) as in Eq. (2):

2 $$ {\text{cf}} = {\text{V}}_{{\text{p}}} + {\text{V}}_{{\text{r}}} {\text{/V}}_{{\text{r}}} $$

### Extraction of BPA

Following the phase separation procedure, BPA in the soluble and colloidal phases was extracted depending on solid phase extraction (SPE) system with ENVI-18 cartridges. Prior to SPE, samples of both phases were acidified and adjusted to pH = 5 by 1 mol/L of formic acid, and then the samples were spiked with 100 ng of standard solution 2 h before commencing with the extraction. All SPE cartridges (500 mg, 6 mL, Supelco) were conditioned with 5 mL methanol and 5 mL ultrapure water. The samples were loaded onto each cartridge slowly and were passed through the cartridges at a rate of around 5 mL per min. The cartridges were then rinsed with 6 mL of methanol/water (30:70, v/v) as well as 6 mL of ultrapure water to eliminate any interferences. Afterwards, the analytes were eluted with 10 mL methanol and the extracts were evaporated to dryness using a rotary evaporator and under a gentle stream of N2, and were then reconstituted with 1 mL of methanol. At last, BPA was analysed using a 6210 Triple Quadrupole liquid chromatography mass spectrometry (LC–MS/MS) with an ESI source following the standardized procedure. The column temperature was maintained at 40 °C and the flow rate of extracts was 0.3 mL/min with volume injection of 5 µL. The analysis was performed at a commercial laboratory.

### Characterization of colloids and particles

Dynamic light scattering (Zetasizer Nano ZS, Malvern) which measures particle sizes between 1 and 1000 nm ranges was employed to calculate the particle size distribution (PSD) in filtered river water samples. The focus was on characterizing colloidal and nano particles in these fractions. The principle of DLS is relatively simple, thermally induced collisions between solvent molecules and the suspended particles trigger the particles in the suspension to undergo Brownian motion. When the particles are irradiated with a laser, the intensity of the scattered light by the particles varies over very brief spans at a degree that depends on particle sizes, smaller particles get further displaced by solution molecules and move about at a more rapid speed. Consequently, this fluctuation in intensity generates Brownian motion’s velocity and ultimately uncovers the size of particles by applying Stokes–Einstein equation.

Furthermore, The Zetasizer was also used for measuring the zeta potential (mV). For small particles in a fluid, there are several techniques for determining the surface charge of particles but they all have mixed views. The most common approach is to find out the electric potential of a particle at a location other than the particle surface, which is someplace in the diffuse layer of the particle. This site is normally called the shear or slipping plane and it relates to the movement of particles in liquids. The electric potential measured at this site is referred to as zeta potential, this parameter is highly significant for nanoparticles and colloids in suspensions. Zeta potential, also known as electrokinetic potential, is valuable in terms of how closely it is linked to particle surface morphology and suspension stability. Zeta potential values normally vary from + 100 mV to − 100 mV. Samples were measured in a 12 mm (PCS1115) Square glass cuvette with cap for 'dip cell' electrophoresis measurement and size and molecular weight measurements. The measured electrophoretic mobilities were converted to zeta potential using the Smoluchowski approach. Zetasizer was used to measure the Ionic strength (mS/cm) as well.

### Water quality parameters

Element analysis was performed on samples for lead (Pb), Zinc (Zn), iron (Fe) and copper (Cu) using ICP-MS after acid digestion following the standard procedures. TSS were quantified using glass fibre filters (0.7 µm) and the turbidity was measured in-situ along with the total dissolved solids, pH, salinity, electrical conductivity, temperature and dissolved oxygen. Additional water quality measurements were performed after transporting the samples to the laboratory including BOD, COD, phosphate, ammoniacal nitrogen (NH_3_–N), oil and grease, *E. coli* and total coliform. All the analysis for water quality parameters were done in accordance to the methods established by APHA in Standard Method Analysis of Water and Waste Water (APHA 2010). Results of water quality analysis can be found in the supplementary (Table S1).

### Quality assurance and quality control

Multiple Reaction Monitoring (MRM) mode was used for quantitative BPA analysis. The linear calibration curves were developed by examining standard solutions ranging between 0.05 and 500 ng/mL, and then followed by applying internal standards. The detection limit and quantification limit (LOD and LOQ) were marked as the corresponding signal-to-noise concentration (S/N) of 3 and 10, respectively. Detection limit and quantification limit of BPA derived from water were 0.2 and 0.3 ng/L respectively. In recovery experiments, a mixture of two working standards containing BPA (1 and 0.1 μg/L) were spiked into 1 L of surface water (n = 3), and further underwent the same process as samples. The recoveries were approximately 93.1% and 118.0% (SD < 5.5%) respectively.

## Results and discussion

### Interactions between colloids and BPA

The aim was to investigate the interactions of colloidal particles with BPA molecules in natural river water. However, as the chemical composition of the colloidal particles in rivers is unknown and is likely to differ and vary from one place to another, test particles in suspensions based on humic acid were used as references to colloidal particles in order to investigate the interaction between colloids and BPA in river water and to illustrate the presence of BPA in the colloidal and soluble phases.

**Figure 2:**
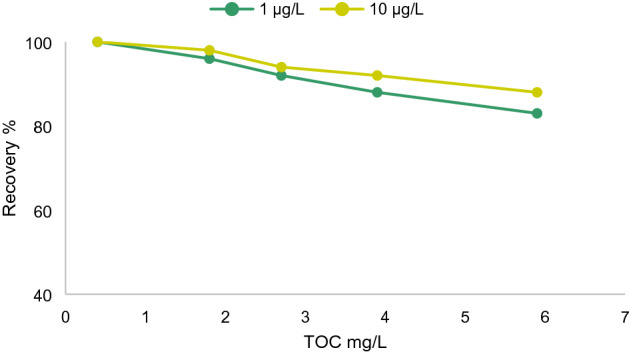
Recovery percentage of BPA from the solution compared to a standard BPA solution and impact of colloidal particles’ concentration on BPA’s extraction.

Colloidal particles and dissolved organic matter in surface water can interact with organic pollutants through numerous adsorption and binding mechanisms, and these interactions have a substantial impact on the transformation and migration of hydrophobic organic pollutants^18^. The effects of humic acid concentration (as a colloidal suspension) on BPA extraction were estimated. Samples containing certain concentrations of humic acid were concocted, and then injected with BPA’s stock solution in two different doses (1 and 10 µg/L). Recovery percentages of BPA from the solution compared to a standard BPA solution are illustrated in (Fig. 2). It is quite noticeable that as the concentration of the colloidal suspension increased, there was an apparent decline in recovery rate, which sagged to about 17% loss opposite the lowest BPA dosage. These results indicated that humic acid as a colloidal suspension in the solution interfered with the recovery rate of BPA molecules.

Furthermore, solid-phase microextraction (SPME) has gained great attention lately in sample preparation since it is a technique that is sensitive, solvent-free, accessible and time efficient. It is also naturally suited for hydrophobic compounds’ extraction in aqueous solutions^19^. The impact of humic acid colloids on BPA in multiple solutions was tested. The main idea was to find out and compare the reactions that take place and their effect on the extraction or recovery of BPA. Two types of SPME experiments were conducted, one was to test the sorption capacity of both humic acid colloids and BPA on the SPME fibre, under the assumption that these two compounds won’t interact effectively in water and will rival each other for the sorption spots on the extraction fibre. The other experiment was to test whether complexes of BPA and humic acid colloids (premixed) will get extracted by the extraction fibre in comparison to free BPA compounds.

Additionally, the binding interactions between colloidal particles (humic acid test suspension) and BPA are highly influenced by the conditions of the solution, since hydrophobic interactions, aromatic stacking and hydrogen bonds are taking place and developing at different temperatures, ionic strengths and pH values. The latter in particular is considered a vital variable that governs and influences the different interactions between dissolved organic matter and pollutants^20^. Depending on pH level, BPA can exist in three different forms in solutions (mono-anion, molecular and dianion) since it carries two phenol groups^21^. Similarly, the configuration of humic acid as a dissolved organic matter is also dissimilar under different pH values^22^. Therefore, our experiment was conducted under a room temperature degree and a neutral pH level, since it is a favourable condition for humic acid particles and BPA to bind and interact and it also enhances BPA’s dissolution in aquatic systems^23^.

**Figure 3:**
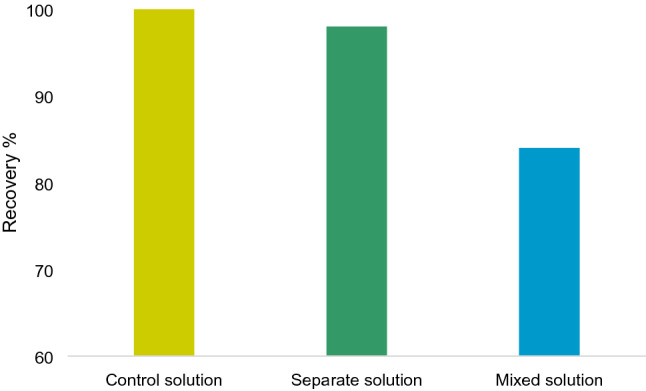
Effect of different mechanisms on interaction between BPA and colloidal particles and extraction efficiency of BPA.

The recovery percentage of BPA in both tests is illustrated in (Fig. 3). There was a noticeable decrease in the recovery percentage in the mixed solution of BPA and humic acid colloids, which dropped off to about 84% compared to the control BPA solution. Conversely, no apparent competition between BPA and humic acid was perceived for extraction, except for an insignificant drop in the recovery rate. These results indicated that the interaction between BPA and humic acid colloids prevented BPA’s sorption onto the SPME fibre, which led to a marked decline in the extraction percentage of BPA from water. Moreover, the likely cause behind this decline was that humic acid colloids were forming compounds or complexes with Bisphenol A that were unextractable by the fibre. Similar outcomes were also reported by Xu et al.^24^ who stated that the presence of humic acid particles inhibited the sorption of Triclosan onto fibrous membranes due to competitive adsorption. These outcomes could also be interpreted as a result of occupying the adsorption sites on the fibre and the blockage of the pore entrance by humic solution.

Moreover, dissolved organic matter substantially influences the transformation and fate of pollutants in water, like binding with hydrophobic organic chemicals (such as BPA) to make up complexes^25^. Some processes contribute to binding interactions between BPA and dissolved organic matter, for instance hydrophobic force, electrostatic interaction, hydrogen bond and aromatic stacking^26^. The binding mechanisms between humic acid and BPA under neutral pH are mostly dominated by hydrophobic forces and hydrogen bonding^23^. This method allowed for accurate determination of BPA as a free substance and in the presence of humic acid.

### Soluble BPA in river water

**Figure 4:**
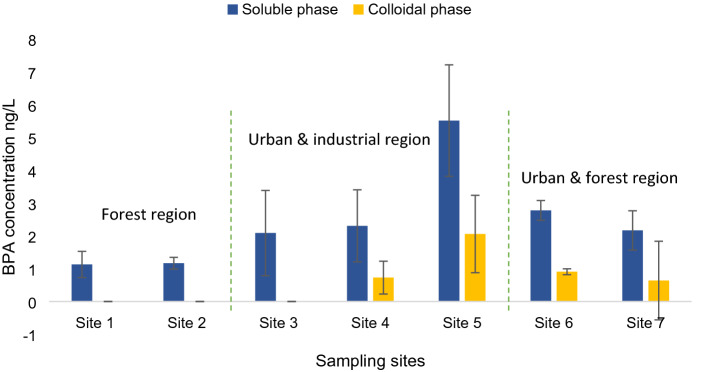
Distribution of BPA in the soluble and colloidal phases in Bentong River.

Bisphenol A was observed in all samples in the study area. The measured concentrations of BPA are shown in (Fig. 4). BPA concentrations varied between 1.13 ± 0.4 and 5.52 ± 1.7 ng L^−1^ in surface water and the highest concentration was registered at site 5 downstream the river inside the district. There are no previous studies in this particular field neither in this river nor in the Semantan River farther downstream so it was not possible to compare and evaluate our results. Bentong River is considered a main water supply for the Semantan water intake that provides millions of people with potable water across four states, which renders this river a critical importance for public safety. Nonetheless, BPA was reported in other rivers in Malaysia; Santhi et al.^27^ detected BPA in 93% of samples taken from Langat River with limits ranging between 1.3 and 215 ng L^−1^ in surface water and between 3.5 and 59.8 ng L^−1^ in tap water. Similarly, Wee et al.^16^ also detected BPA in all collected samples in Langat River with a registered maximum concentration of (8.24 ng L^−1^). Duong et al.^28^ also reported levels of BPA (7.4–10.8 ng L^−1^) at Salut River in Sabah, Malaysia.

Forest trees and plants surrounded site 1 in the study area and this sampling station is located upstream before the river enters the main population and industrial areas. This site registered the lowest value of BPA (1.13 ± 0.4 ng L^−1^) among other sites. There were no industries upstream and around site 1, although direct emissions from villages and scattered houses situated close by were spotted when surveying the study area. Beyond this point, the river enters the populated area and receives wastewater effluent from several sewage treatment plants. The composition around sites 2 and 3 is mostly houses and urban environments along with few small industries. However, Bentong hospital is located upstream ahead of these two sites. Site 2 registered a rather similar value to site 1 (1.17 ± 0.18 ng L^−1^), while the concentration increased noticeably at site 3 (2.09 ± 1.3 ng L^−1^). Higher levels of BPA were recorded at sites 4 and 5 (2.31 ± 1.14 and 5.52 ± 1.7 ng L^−1^ respectively) which are located farther down the river and thus receive wastewater effluent not merely from upstream but from the adjacent areas as well, particularly site 5 which is situated near the boundary of Bentong District. Industry practices around these last two sampling stations are comprised of rubber factory, paper factory, steel towers manufacturing, chemicals manufacturing, two car service companies and a textile mill. BPA levels at sites 6 and 7 (2.78 ± 0.32 and 2.17 ± 0.64 ng L^−1^ respectively) declined slightly after the river departed the main inhabited zone, site 6 is about 8 km in distance from site 5 and both receive effluent from scattered points. The significance of the last two sites is that they precede the Semantan water intake, which is positioned approximately 10 km from site 7. Consequently, it is not improbable that BPA can be found in the abstracted raw water.

In 2012, the Department of Environment (DOE) in Malaysia had pinpointed a total 1,662,329 point sources that contribute to water pollution all over Malaysia, comprised of 9883 STPs, 4595 manufacturing industries, 754 animal farmhouses, 865 wet markets, 508 agro-centred practices, and 192,710 food service enterprises^29^. Wee et al.^16^ stated that urban and Industrial effluents from STPs are considered the main sources of BPA in drinking water supplies in Malaysia. However, BPA concentrations can be considered low in comparison with other results reported in the region including in Malaysia. This can be attributed to absence of major industries along Bentong river reach, and potentially to the strict regulations on BPA’s use which prohibited excessive application of BPA^16^. Similar low levels of BPA were also reported in Malaysian rivers such as Langat River^16,27^ as well as Salut River^28^. Due to the its wide-ranging use and physicochemical characteristics, BPA has been detected in some mariculture production in Malaysia, registering the highest values (0.023–0.322 ng g^−1^) among other plasticizers measured in fish muscle^30^. Additionally, BPA’s demand globally is projected to rise to around 10.6 MMT by 2022^31^. This continuous use could result in increasing BPA’s presence all over water systems.

**Figure 5:**
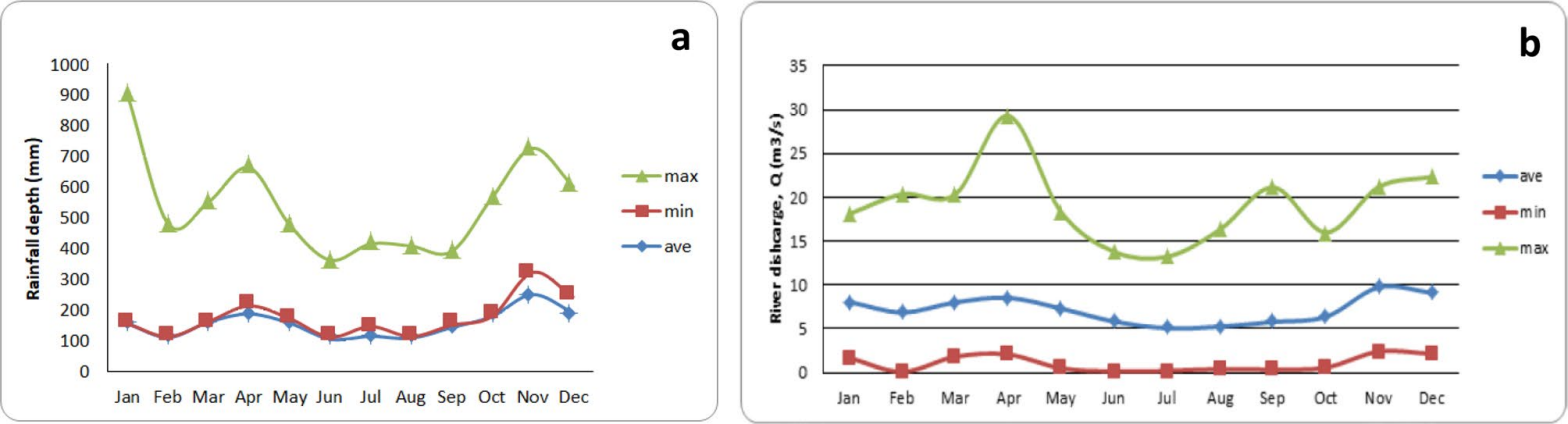
(**a**) Monthly distribution of total rainfall and (**b**) river discharge.

Additionally, it is well known that seasonal variation and environmental factors impact BPA’s presence in river water. Meteorological rainfall data across Bentong river catchment revealed that total rainfall was highest in November (Fig. 5A) with an average temperature of 27 °C during that period. River flow across the undertaken section averaged 25.3 m^3^ s^−1^ with November again registering the highest flow rate (Fig. 5B). Although seasonal variation of BPA in Bentong River can’t be adequately demonstrated with one set of samples, these figures did offer some insight as to the overall condition in Bentong river. Warm seasons coupled with ample rainfall and increased flow discharge may largely lead to dilution of BPA. Normally, biodegradation rates are faster at high temperature degrees^32^, and adsorption by microalgae, which blooms in warmer periods, can lead to BPA biotransformation in water^33^. High water temperatures can also enhance sorption of BPA by particulate and colloidal organic carbon^33^. During heavy rainfall periods, which correspond to our case in Bentong, WTPs and retention basins become overloaded causing untreated wastewater to spill straight into receiving water. Moreover, as rainfall scour across impermeable tops, it collects sediments, nutrients and further contaminants and deposits them into the closest watercourse^34^. Both of these incidents can increase BPA’s level in river water during heavy rain seasons, which overall might cause higher levels of BPA in summer in comparison with other seasons.

### BPA in the colloidal phase

Aquatic colloids are ubiquitous in natural aquatic environments with sizes ranging from 1 nm to 1 µm and they can play a major role in regulating the behaviour and fate of pollutants in water. BPA concentrations in the colloidal phase are shown in (Fig. 4). Undoubtedly, BPA’s concentration was extremely lower in the colloidal phase, but it remains an indication of their high biological availability. Corresponding to its soluble phase, the highest BPA concentration was detected in the colloidal phase at site 5 at 2.06 ± 1.18 ng L^−1^, followed by site 6 at 0.91 ± 0.09 ng L^−1^.

Colloids’ proportion in the traditionally dissolved phase was calculated to assess its contribution in adsorbing and capturing BPA in water. Generally, the particulate phase in water and specifically the colloidal phase has a substantial part in controlling pollutants’ transport and fate in water^35^. Therefore, analysing the distribution of pollutants among the different phases or fractions in water offers a valuable insight into these processes. The proportion of BPA present in the colloidal phase was estimated using Eq. (3):

3 $$ \Phi = {\text{C}}_{{\text{c}}} {/}\left( {{\text{C}}_{{\text{c}}} + {\text{C}}_{{{\text{water}}}} } \right) \times {1}00 $$

where C_c_ and C_water_ are BPA’s concentration in the colloidal and soluble phases respectively. Φ represents the fraction or percentage of BPA adsorbed onto colloids. The spatial distribution of BPA in relation to land cover is also shown in (Fig. 4).

**Figure 6:**
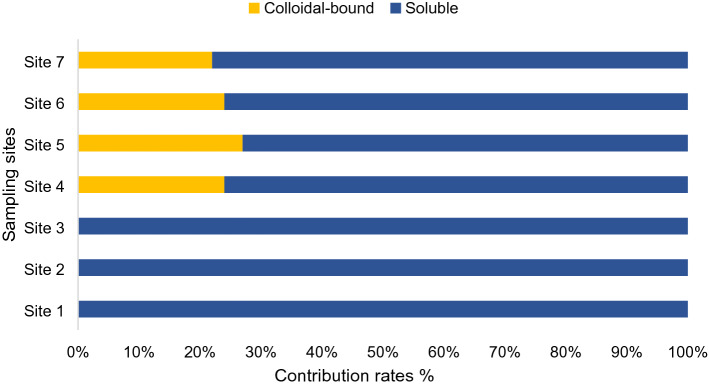
Proportion of BPA in soluble and colloidal phases in Bentong River.

BPA’s distribution in the colloidal and soluble phases is depicted in (Fig. 6). The fraction of BPA molecules adsorbed onto colloidal particles varied from 0 to 24% in Bentong river. These levels, as small as they may seem, imply that colloids can in fact function as a sink or milieu for pollutants in general including EDCs, and reflect to some extent BPA’s physiochemical properties. Adsorption to suspended particulate matter wasn’t measured in this research and it is completely feasible that BPA could be attached to them too and this case was reported previously as well^36–38^. Nonetheless, colloids and smaller particles possess high contaminant reactivity and higher sorption capacity in comparison with larger particles and solids^10^. Unfortunately, data sets on emerging contaminants’ distribution within the colloidal phase including BPA are somewhat scarce, therefore it’s a tad tricky to equate these findings with others. Still, similar work in this field has been conducted which align with most of what has been stated beforehand. Kalmykova et al.^9^ reported that PAHs were attached predominantly to smaller colloidal particles in contrast to larger particulate matter. Similarly, Prater et al.^39^ stated that colloidal particles demonstrated a robust sorption capacity for estrogenic compounds, and have a tendency to shield and protect them from degradation throughout transportation. Duan el al.^10^ also illustrated colloids’ influence on pollutants stating that 4–45% of analysed pharmaceuticals were associated with colloids, and that contaminants had exhibited greater affinity towards colloidal particles compared to suspended particulate matter.

Furthermore, according to the various values reported in literature, BPA has a moderate octanol/water partition coefficient (K_ow_) value that normally varies between (2.2 and 3.8). This dimensionless octanol–water partition coefficient is normally used to predict the potential environmental fate and bioaccumulation of BPA^40^. The K_ow_ is a measurement of the lipophilicity of a compound and is defined as the ratio of the equilibrium concentrations of a dissolved substance in octanol and water. Chemicals with a log K_ow_ higher than 4 aren’t normally very soluble in water (hydrophobic), while those with a log K_ow_ less than 1 are highly soluble in water (hydrophilic)^41^. Given this information, it is relatively fitting to label BPA as having a moderate potential to relocate from the water phase into a solid phase. In other words, BPA has a moderate tendency for sorption to particles or bioaccumulation^42^. Consequently, this all suggests that BPA’s association with colloids is taking place due to hydrophobic interactions. Even though BPA was detected broadly in the soluble phase in all samples and it had a rather low contribution ratio from the colloidal phase, still, a moderately high log K_ow_ typically propels compounds to bind easily with colloids and small particles, causing relatively higher concentrations in the colloidal phase compared to the soluble phase^43^. This information must be considered all through water quality standards’ development for pollutants in aquatic ecosystems or any development of environmental risk assessments.

Although BPA is not precisely a persistent organic pollutant owing to its short half-life and susceptibility to degradation, it is frequently grouped together with other persistent organic pollutants due to the continuous exposure and introduction of this chemical into the aquatic system. Despite its short half-life, BPA is frequently found, particularly lately, in surface water, groundwater, stormwater, sediments and even in urine at a relatively persistent level which bears behavioural resemblance to chemicals with much longer half-life^44^. Moreover, BPA attached to colloids and particles of diverse natures is subjected to transformations and reactions that can lead to the formation of intermediate compounds.

### Colloids characterization in water

**Figure 7:**
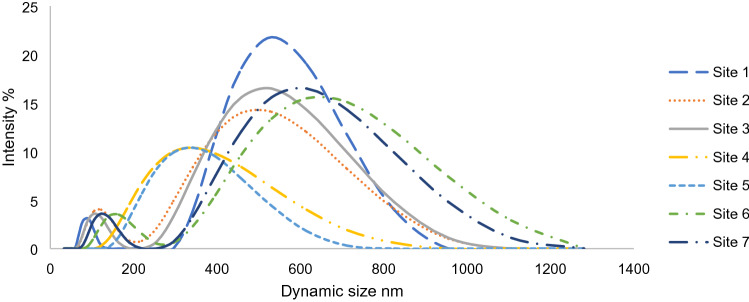
Particle size distribution (PSD) of colloidal particles in Bentong River samples.

Generally, colloids are defined as mixtures of heterogeneous particles with various structures, forms, sizes, surface chemistry and chemical composition^45^. Particle size distribution displayed a majority of particles smaller than 1.8 µm, although the accumulated average diameter varied among samples. Zetasizer measurements indicated nanoparticles’ presence in some samples, however a specific particle size distribution was difficult to obtain since the samples were multifarious as demonstrated in (Fig. 7).

The size distribution of particles in surface water samples exhibited a distinctive natural water outline, where the general trend for particle numbers is to swiftly decline as the particle diameter increases^46^. The majority of the samples registered a presence of two particle peaks, which corresponded to increasing particle intensity. Particle size distribution analysis showed two main particle groups, first one had a size range between 0.4 and 0.9 µm and the other group between 0.09 and 0.18 µm with very slight scattering (Fig. 7). Moreover, the first group had a higher percentage of volume and intensity compared to the second group, indicating higher particle concentration in the first size group.

**Figure 8:**
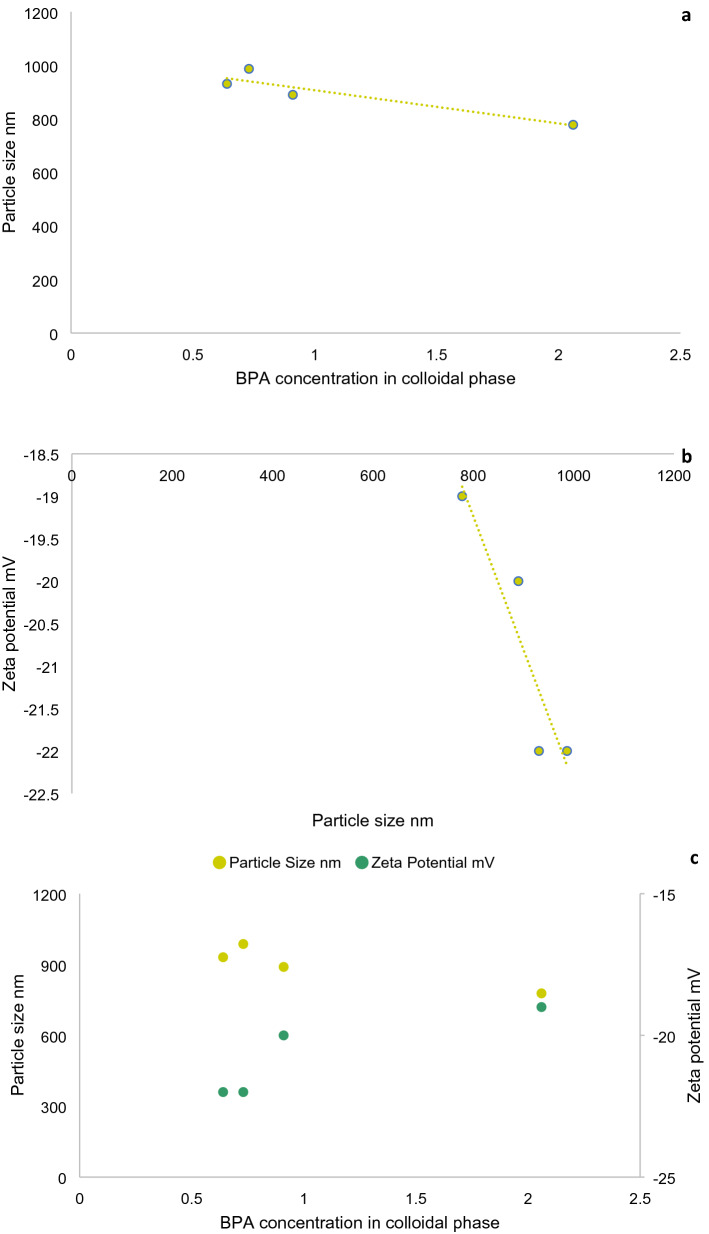
Correlations between (**A**) BPA conc. in the colloidal phase and PSD, (**B**) PSD and zeta potential and (**C**) BPA, PSD and zeta potential.

Generally, there is an inverse relationship between particle size distribution and zeta potential for nanoparticles. Zeta potential for all samples was negative, ranging between − 25 to − 18 mV, and these negative values suggested instability of particles^47^. Consequently, due to these low zeta potential values, the particles tend to cluster which means that they are susceptible to coagulation and flocculation. These negative values are consistent in particles that contain organic acid compounds such as humic acid and fulvic acid which had been reported previously in several studies^48^. A positive correlation was observed between the zeta potential and particle sizes (Fig. 8), which could be explained by the extended (DLVO) theory. Van der Waals attraction forces surrounding the particle are low compared to repulsion forces when zeta potential value is high, which yields particles of small sizes and dispersion stability^45^.

Additionally, pollutants’ transport (e.g. metallic elements and pesticides) may be facilitated by small sized particles, which indicates that colloids may possess greater sorption capacities compared to larger particles. Noticeably, there was a negative correlation between BPA concentrations in the colloidal phases and colloids hydrodynamic sizes (Fig. 8). This implied that particle sizes, particularly colloids could be a significant factor controlling or influencing BPA’s sorption in aquatic environments.

### Colloidal component in river water

Colloids’ dry weight average concentration in Bentog River samples was 12.7 mg/L. This was much higher than the colloidal organic carbon (COC) average of 2.25 mg/L derived from DOC analysis. This discrepancy isn’t that surprising as colloids’ components normally quantified via dry weight may consist of both inorganic and organic material such as aluminosilicates (clays), silica, aluminum oxides, iron oxides, manganese oxides and oxyhydroxides. Wilding et al.^49^ suggested that colloid organic matter (COM) concentration in a certain solution is nearly 2.7 times that of colloidal organic carbon. Thus, colloid organic matter equalled 6.08 mg/L of measured colloidal organic carbon. Plus, the ratio of COM to colloids’ concentration was estimated to be 48%, suggesting that about 52% of colloids are basically inorganic in nature. Several methods have been suggested to quantify the COC percentage in total DOC of samples, among them is Eq. (4):

4 $$ \% {\text{COC}} = \left( {{\text{OC}}_{{\text{r}}} - {\text{OC}}_{{\text{p}}} } \right){/}({\text{cf}} \times {\text{DOC}}) $$

The COC percentage in total DOC was around 14%. This value is slightly lower than those presented in other research works. Wilding et al.^49^ found it to be at 16% in Longford Stream, UK. While Yan et al.^50^ indicated that there is at least 8% of COC in total DOC that can go up to 12–19% depending on the cut-off filter pore size of either 0.4/0.45 or 0.2/0.22 µm. Moreover, Benner and Amon^51^ estimated that low molecular weight OC comprised 77% of total OC in oceans. This relatively lower percentage of COC in Bentong River compared to other values is attributed perhaps to the high content of soil run-off material washed over during intense rainfalls that occur regularly in Malaysia, which results in various organic and inorganic soil elements in the riverine system.

### Determining the enhanced capacity

Passive dosing technique is an established procedure for accomplishing constant exposure in aqueous solutions^52^. Numerous methods have been stated before to saturate a hydrophobic chemical onto a certain passive dosing polymer^53^. This technique was applied to illustrate the sorption of BPA onto natural colloidal particles by releasing BPA from a silicone membrane into river water samples. BPA was quantified after achieving equilibration state. The enhanced capacity and free fraction of BPA were quantified, BPA’s concentration quantified in equilibrated pure water acted as a reference to the freely dissolved BPA in the solution, whereas the total concentration of BPA quantified in the equilibrated solution was referred to as the dependent variable. Then, the free fraction (ff) was determined as the proportion between the concentrations in the equilibrated pure water to that in the solution as in Eq. (5):

5 $$ {\text{ff}} = {\text{C}}_{{{\text{water}}}} {\text{/C}}_{{{\text{solution}}}} $$

After that, the enhanced capacity was determined as the ratio of the solution to that of pure water, a ratio above 1 implies to the sample’s capacity to containing higher levels of a substance compared to the reference water^54^ as in Eq. (6):

6 $$ {\text{E}} = {\text{C}}_{{{\text{solution}}}} {\text{/C}}_{{{\text{water}}}} $$

The enhanced capacity (E) measures a solution’s capacity to hold hydrophobic organic chemicals relative to clean water. This concept is relatively similar to that of “solubility enhancement” though they shouldn’t be mixed together. The enhanced capacity is different in that it may be quantified at or below the saturation level of hydrophobic organic chemicals. Whereas the solubility enhancement is only applied to and measured at the saturation level of these chemicals^54^.

Water samples from Bentong River were used as a medium for this assessment. The samples were filtered with 0.2, 0.7 and 1.2 µm filters, producing three samples overall. The reasoning behind using three types of filters was to find out whether the enhanced capacity varies with different contents of colloidal particles in the samples, considering that each filter would allow a certain amount of colloidal size to pass through its pores. Afterwards, passive dosing was applied to the filtrated samples where BPA was loaded onto a silicone membrane and then immersed into the samples. equilibrium was achieved within 24 h.

**Figure 9:**
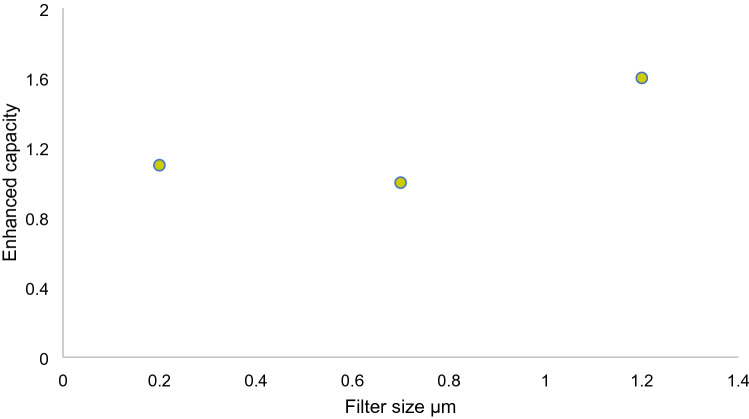
Enhanced capacity in river water samples separated by three filters (1.2 µm, 0.7 µm and 0.2 µm).

The results showed that all three samples had the capacity to contain higher BPA molecules than the reference water (Fig. 9). The highest enhanced capacity ratio was registered in the 1.2 µm filtered sample. Noticeably, the enhanced capacity ratio in the samples declined when particles content lessened due to filtration, which in turn implied to the insufficiency of sorbing surfaces for BPA. The samples containing nanoparticles (0.7 µm and 0.2 µm) did not reflect a high ratio of holding capacity despite the fact that smaller particles have a high contaminant reactivity and also a high tendency for sorption due to their unique characteristics^9^. The reason behind this discrepancy may be attributed to a lack of nanoparticles to begin with (> 1 µm) in the collected samples which have the ability to adsorb BPA compounds.

The enhanced capacity is particularly significant in regards to the environmental fate of hydrophobic organic chemicals in general. Colloid-facilitated transport of hydrophobic organic chemicals through soil macropores has been identified as a potential transport mechanism toward drainage pathways and groundwater^55^. Furthermore, the enhanced capacity of particles can be accurately calculated and connected to the dissolved organic matter present in the sample. The main advantage of this indicator is that it can be measured below the saturation level, which offers novel options for dealing with small environmental levels and related mix constituents^54^.

### Determining the sorption coefficient (K_coc_)

**Figure 10:**
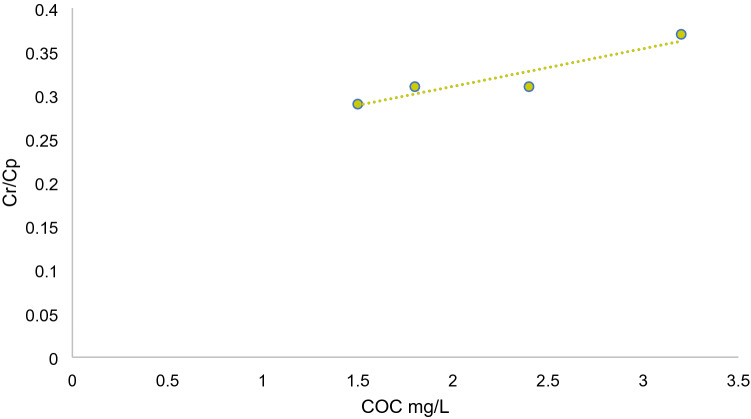
Ratio of BPA concentration in retentate (C_r_) to permeate (C_p_) in relation to colloidal organic carbon concentrations.

The K_coc_ value was calculated from Eq. (7), and are shown with *y*-intercepts, correlation coefficients, and *p* values of best-fit lines in (Fig. 10).

7 $$ {\text{C}}_{{\text{r}}} {\text{/C}}_{{\text{p}}} = {1} + {\text{K}}_{{{\text{coc}}}} \left\{ {{\text{COC}}} \right\} $$

Results demonstrated a fine linear relationship with a K_coc_ *p *value of 0.04, which suggested significant relationships. Sorption coefficient value was estimated at 6.8 × 10^3^ (mL/g), which is similar to what Liu et al.^56^ and Yan et al.^13^ stated, but rather smaller compared to the values reported by Zhou et al.^57^ and higher than what Huang et al.^58^ reported. The differences can be accredited to the diverse characteristics of colloids in natural streams from one place to another, also to various environmental elements e.g. colloidal organic content and pH^10^. Nevertheless, these outcomes are significant as they identify riverine colloids as a possible reservoir for BPA in aquatic environments^59^, and since colloidal particles possess large surface areas, their adsorption with BPA could be attained within a short period^56^. Furthermore, organic pollutants’ sorption onto colloids is considerably influenced by the origin and source of water. There is an inclination that colloidal particles in natural water sources have a higher sorption capacity in comparison with those found in wastewater. Yan et al.^60^ mentioned that emerging organic pollutants’ sorption capacity to low molecular weight colloids was much higher compared to high molecular weight colloids. Moreover, he stated that most high molecular weight colloids could be removed through degradation and aggregation in WWTPs, which could lead to a dominant role by low molecular weight colloids in natural water and increase their sorption capacity of emerging organic pollutants (such as BPA) in natural water.

In addition, Huang et al.^58^ estimated the partition coefficient for SPM-bound (K_poc_) BPA and colloidal-bound (K_coc_) BPA, Log K_poc_ was valued at 4.59, while log K_coc_ was a little higher at 4.87. This suggested that aquatic colloids, compared to SPMs, were more prominent carriers of BPA in surface water.

### Relationship between BPA and water quality parameters

Water samples from Bentong district showed that the highest BPA concentration was detected downstream near the confluence of Bentong River with some of its tributaries. There were large amounts of treated and untreated urban and industrial wastewater effluents discharged into the river. Gong et al.^61^ stated that BPA’s primal sources are normally linked with the vicinity of urban and industrialized activities. Shi et al.^62^ also mentioned that BPA concentrations were high in the Yangtze River Estuary which received high loads of domestic sewage compared to industrial wastewater.

**Figure 11:**
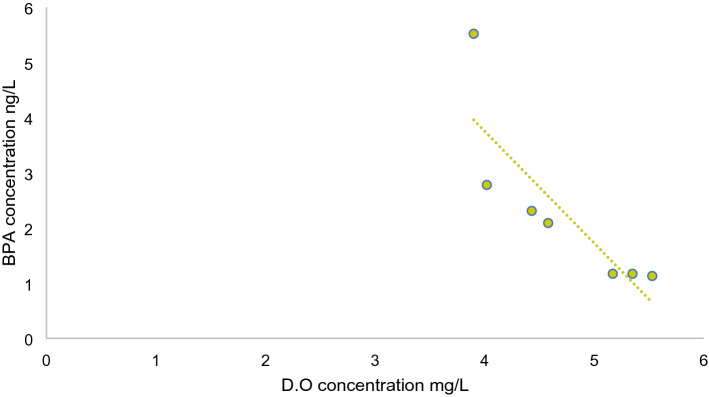
Correlations between dissolved oxygen concentration and BPA in Bentong River.

Water quality parameters were correlated with BPA in an attempt to identify some existing relations between them. Although the sample size was small and accurate correlations normally require sufficient data for better representation, our data still displayed significant outcomes. BPA was positively correlated with BOD, COD and NH_3_–N which are indicators of organic contamination. This could imply that BPA and these organic compounds were released concurrently. Conversely, BPA had negative correlations with dissolved oxygen and pH, indicating that BPA might not be readily degraded with low vales of pH and dissolved oxygen (Fig. 11). Undoubtedly, higher content of dissolved oxygen along with suitable microorganisms for BPA degradation would lead to lower concentration of BPA detected in water^63^. Additionally, Zeng et al.^64^ stated that BPA has higher solubility at alkaline pH ranges which was attributed to low adsorption capacity onto sediments. pH values in our study were not in the alkaline range and BPA concentration was not as high compared to other studies, which would suggest that water’s pH was a limiting factor for BPA’s presence. BPA was also positively correlated with NH_3_–N in river water. High content of NH_3_–N in water would require a great oxygen demand for bacteria to convert or ammonia to nitrate, which would lead to oxygen reduction in water and thereby affecting biodegradation of different compounds including BPA^65^.

As mentioned previously, available data suggest that BPA does not persist significantly under aerobic conditions^66^. However, this compound degrades only slowly or does not degrade at all under low or no oxygen conditions. Thus, this presents a significant issue in treating and removing BPA from wastewater and sludge where oxygen levels are immensely low. Moreover, even with its relative degradable nature, BPA can persist in water for long periods due to sorption on particles and slow rate degradation^65^.

Additionally, the massive usage of heavy metals in numerous industries currently enriches industrial and municipal wastewater and even stormwater with heavy metals and organic substances. Studies on the interaction and fate of BPA with the presence of heavy metals and organic substances are vital to investigate any adverse effect of BPA on living organisms^67^. BPA commonly coexists with heavy metals and active organic compounds in contaminated surface water, and the photochemical behaviour of metallic ions in water affects the transformation of organic pollutants. Heavy metals were analysed in our study for lead (Pb), Zinc (Zn), iron (Fe) and copper (Cu) and were detected with mean concentrations of (6.5, 56, 1004, 8.4 ppb) respectively. Complexes of Fe (III)-Ox were found to induce photooxidation of BPA in water at pH values between 3 and 8 under radiation of UV light, and this process was highly dependent on Fe concentration^68^. In contrast, Kim and Nicell^69^ stated that Fe (III), Cu (II) and halogens chloride and fluoride noticeably suppressed BPA conversion in water as well as other compounds such as cyanide. Gonzalez-Rey et al.^70^ stated that heavy metals’ coexistence with endocrine disrupting compounds (including BPA) has an additional impact on the endocrine system.

### Risk quotient and environmental implications

Due to the increasing wide detection and biological availability of BPA in surface water, the continuous exposure of aquatic creatures to this compound may well result in some hazards to aquatic environments. Therefore, a risk assessment for BPA was conducted according to available toxicity figures for fish and algae to illustrate their potential impact. Risk Quotient (RQ) method was employed to evaluate the ecotoxicity of BPA on fish and algae in water^71^. Risk level ranking was done following the criteria (High risk 1 < RQ; medium risk 0.1 < RQ < 1; low risk 0.01 < RQ < 0.1) set by Liu et al.^32^. The calculated RQ values for BPA were all under 0.01, thus the risk posed by BPA for sensitive aquatic creatures was low. Generally, Plasticizers (such as BPA) and hormone groups have a higher risk probability in comparison with other emerging pollutants due to their stability in water having a rather low solubility coefficient (around 3.32)^16^. Moreover, occurrence of BPA is connected with additive, synergistic and antagonistic impacts on changing gene expressions which can cause cell proliferation in small dosages^72^. Also, potential behavioural alterations and developmental toxicity are associated with emerging pollutants such as BPA^73^. According to prior studies, adverse effects may well befall on the growth status of *Raphidocelis subcapitata*, in addition to hatching success of fish embryos and body pigmentation due to BPA association^74^.

The Semantan water intake situated downstream of Bentong River was in large the principal motive behind this particular study. Also, information scarcity and lack of concern with relation to endocrine disrupting compounds in general and BPA within Bentong River were among the reasons that added extra importance to monitoring and assessment efforts across this water resource. Colloidal particles tend to form a suitable medium for BPA’s adsorption due to their high surface area and are capable of carrying pollutants to long distances increasing their distribution throughout the aquatic system. This study asserted BPA’s presence in river water both in soluble state and attached to colloidal particles. Although Bentong district can’t be described as an industry laden area, there are specific industries within that deal with BPA and its analogues consistently which exposes the river and puts it at risk of higher BPA inputs. Rubber manufacturing is one of the prominent industries in Bentong district that have undoubtedly increased its production intensely with the high demand for gloves and masks in light of COVID-19 pandemic spread all over Malaysia. Furthermore, These BPA levels are bound to increase with large-scale development plans taking place throughout the state that will certainly increase the pollution load on the river. Soluble BPA didn’t seem to linger throughout this river section and its levels declined downstream which highlights the significance of particle attachment for displacement. The extent of colloidal-bound BPA’s impact on the water intake downstream and on aquatic life in general is believed to be minor in light of the levels registered in this particular river stretch, but it is by no means a conclusive outcome and more work should be carried out on a regular basis.

## Conclusion

The presence and distribution of BPA in the colloidal and soluble phases in Bentong River were investigated. BPA had a detection rate of 100% in the soluble phase registering a maximum level of 5.52 ng L^−1^. In the colloidal phase, BPA had a maximum level of 2.06 ng L^−1^ with a 57% detection rate, and the adsorption percentage of BPA on colloids was estimated at 24%. Colloids exhibited a capacity for binding with BPA as a moderate hydrophobic pollutant, consequently, the influence of colloids on the environmental transformation and fate of BPA and other compounds should be elucidated further. Moreover, PSD of colloids indicated the presence of two major groups of particles, between the size range of (0.4–0.9 µm) and (0.09–0.18 µm) with very slight scattering. Zeta potential was found to be negative, ranging between − 25 and − 18 mV, suggesting instability of particles and indicating the particles’ tendency to cluster as well as to coagulate and flocculate. Furthermore, BPA showed negative correlation with DO and a positive one with NH_3_–N and organic content which indicated although not decisively the source of BPA discharge into the river. RQ assessment showed low levels of risk which corresponded well with the results at hand in this particular river system. Nevertheless, colloids are heterogeneous particles with varying compositions and more information on the interaction between colloids and BPA as well as other substances in the aquatic environment should be pursued further. Also, the influence of the environmental and hydrological factors on colloids behaviour requires more illustration in the future.

## Supplementary information

Supplementary Information.
