# Supplementary material for: Occurrence, environmental implications and risk assessment of Bisphenol A in association with colloidal particles in an urban tropical river in Malaysia
Source: Sci Rep. 2020 Nov 23;10:20360. doi: 10.1038/s41598-020-77454-8 (PMC7683680; doi:10.1038/s41598-020-77454-8)
Supplement: Supplementary file 1 — Supplementary Information. [file 41598_2020_77454_MOESM1_ESM.pdf]

Occurrence, environmental implications and risk assessment of Bisphenol A in association with colloidal particles in an urban tropical river in Malaysia

Zakariya Nafi' Shehab [a], Nor Rohaizah Jamil [a, *], Ahmad Zaharin Aris [a]

Table S1. Water Quality Parameters at 20 sampling sites along Bentong River

Parameter/station	Unit	1	2	3	4
pH (In-Situ)	-	6.69	6.6	6.58	6.39
Temperature (in-Situ)	°C	27.3	29.2	26.3	24.4
Dissolved Oxygen (In-Situ)	mg/1	3.75	3.47	4.53	4.74
*Salinity (In-Situ)	ppt	0.02	0.02	0.02	0.01
Conductivity (In-Situ)	p.s/cm	42.9	55.3	54.6	38
Turbidity	NTU	9.35	12.13	8.35	4.36
Chemical Oxygen Demand	mg/1	71	63	42	58
Biochemical Oxygen Demand @ 20°C,5 days**	mg/1	10	6	7	4
Total Suspended Solids	mg/1	30	20	20	10
Ammoniacal Nitrogen	mg/l	1.12	0.56	0.28	0.42
*Nitrate	mg/1	ND<0.01	ND<0.01	ND<0.01	0.03
Sulphate	mg/l	8.28	13.73	15.05	6.48
Total Coliform	CFU/100ml	27000	22000	26000	30000
Total Dissolve Solid (In-Situ)	g/l	3430	50.86	137.1	46.52
Oil & Grease	mg/l	ND<1.0	ND<1.0	ND<1.0	ND<1.0
Phosphate	mg/1	1.14	ND<0.10	0.21	0.42
E-Coli	CFU/100ml	3100	7200	6900	1400

Parameter/station	Unit	5	6	7	8
pH (In-Situ)	-	6.62	6.51	6.48	6.59
Temperature (in-Situ)	°C	26.3	25.8	25.9	26.5
Dissolved Oxygen (In-Situ)	mg/1	5.45	5.7	5.4	5.84
*Salinity (In-Situ)	ppt	0.03	0.02	0.02	0.02
Conductivity (In-Situ)	p.s/cm	72.1	47.8	48.6	52.9
Turbidity	NTU	7.05	5.76	7.47	15.19
Chemical Oxygen Demand	mg/1	79	63	54	42
Biochemical Oxygen Demand @ 20°C,5 days**	mg/1	7	2	3	4
Total Suspended Solids	mg/1	88	12	24	96
Ammoniacal Nitrogen	mg/l	0.28	0.28	0.14	0.28
*Nitrate	mg/1	0.04	ND<0.0 1	ND<0.0 1	ND<0.01

Sulphate	mg/l	13.98	24.93	6.06	5.15
Total Coliform	CFU/100ml	6300	32000	23000	6900
Total Dissolve Solid (In-Situ)	g/l	796.5	841.8	43.27	36.74
Oil & Grease	mg/l	1	ND<1.0	ND<1.0	ND<1.0
Phosphate	mg/1	0.55	1.54	0.64	ND<0.10
E-Coli	CFU/100ml	1700	7100	6400	3000

Parameter/station	Unit	9	10	11	12
pH (In-Situ)	-	6.54	6.6	6.54	6.53
Temperature (in-Situ)	°C	25.3	28	25.9	27.5
Dissolved Oxygen (In-Situ)	mg/1	5.94	3.39	4.84	3.35
*Salinity (In-Situ)	ppt	0.02	0.03	0.03	0.02
Conductivity (In-Situ)	p.s/cm	45.5	72.7	78.1	56.9
Turbidity	NTU	10.49	9.47	9.91	25..96
Chemical Oxygen Demand	mg/1	92	79	63	71
Biochemical Oxygen Demand @ 20°C,5 days**	mg/1	7	7	2	7
Total Suspended Solids	mg/1	42	48	48	52
Ammoniacal Nitrogen	mg/l	0.14	0.56	1.12	0.84
*Nitrate	mg/1	0.01	0.154	0.09	0.89
Sulphate	mg/l	3.09	16.55	2.58	32.49
Total Coliform	CFU/100ml	34000	32000	30000	28000
Total Dissolve Solid (In-Situ)	g/l	73.74	45.59	83.29	31.63
Oil & Grease	mg/l	ND<1.0	ND<1.0	ND<1.0	ND<1.0
Phosphate	mg/1	ND<0.10	0.81	0.09	ND<0.10
E-Coli	CFU/100ml	5900	2500	5800	2000

Parameter/station	Unit	13	14	15	16
pH (In-Situ)	-	6.36	6.25	6.42	6.6
Temperature (in-Situ)	°C	26	24.5	25.9	28.2
Dissolved Oxygen (In-Situ)	mg/1	4.91	5.37	4.98	3.75
*Salinity (In-Situ)	ppt	0.01	0.01	0.01	0.03
Conductivity (In-Situ)	p.s/cm	28.5	24.6	28.21	67
Turbidity	NTU	4.52	2.99	10.74	82.05
Chemical Oxygen Demand	mg/1	40	20	80	64
Biochemical Oxygen Demand @ 20°C,5 days**	mg/1	6	6	4	7
Total Suspended Solids	mg/1	20	14	40	96
Ammoniacal Nitrogen	mg/l	0.56	0.28	1.12	1.96
*Nitrate	mg/1	0.08	0.09	ND<0.01	ND<0.01
Sulphate	mg/l	0.973	12.01	37.62	ND<1.0
Total Coliform	CFU/100ml	8000	7300	8700	8100
Total Dissolve Solid (In-Situ)	g/l	31.13	13.54	213.5	36.21
Oil & Grease	mg/l	ND<1.0	ND<1.0	ND<1.0	ND<1.0
Phosphate	mg/1	ND<0.10	0.39	ND<0.10	ND<0.10

E-Coli	CFU/100ml	1600	2000	2500	2200
Parameter/station	Unit	17	18	19	20
pH (In-Situ)	-	6.69	6.71	6.68	6.65
Temperature (in-Situ)	°C	29.3	28.8	28	28.5
Dissolved Oxygen (In-Situ)	mg/1	3.64	3.53	3.23	3.44
*Salinity (In-Situ)	ppt	0.03	0.04	0.04	0.04
Conductivity (In-Situ)	p.s/cm	71.6	87.26	88.6	88.4
Turbidity	NTU	42.47	42.45	36.4	32.82
Chemical Oxygen Demand	mg/1	40	72	48	56
Biochemical Oxygen Demand @ 20°C,5 days**	mg/1	4	7	7	4
Total Suspended Solids	mg/1	92	76	72	52
Ammoniacal Nitrogen	mg/l	0.28	1.26	1.68	0.56
*Nitrate	mg/1	ND<0.01	ND<0.01	ND<0.01	ND<0.01
Sulphate	mg/l	25.24	13.64	6.76	22.25
Total Coliform	CFU/100ml	18000	11000	24000	19000
Total Dissolve Solid (In-Situ)	g/l	42.1	54.46	43	71.68
Oil & Grease	mg/l	ND<1.0	ND<1.0	ND<1.0	ND<1.0
Phosphate	mg/1	ND<0.10	0.22	0.29	ND<0.10
E-Coli	CFU/100ml	1900	3500	7600	5600

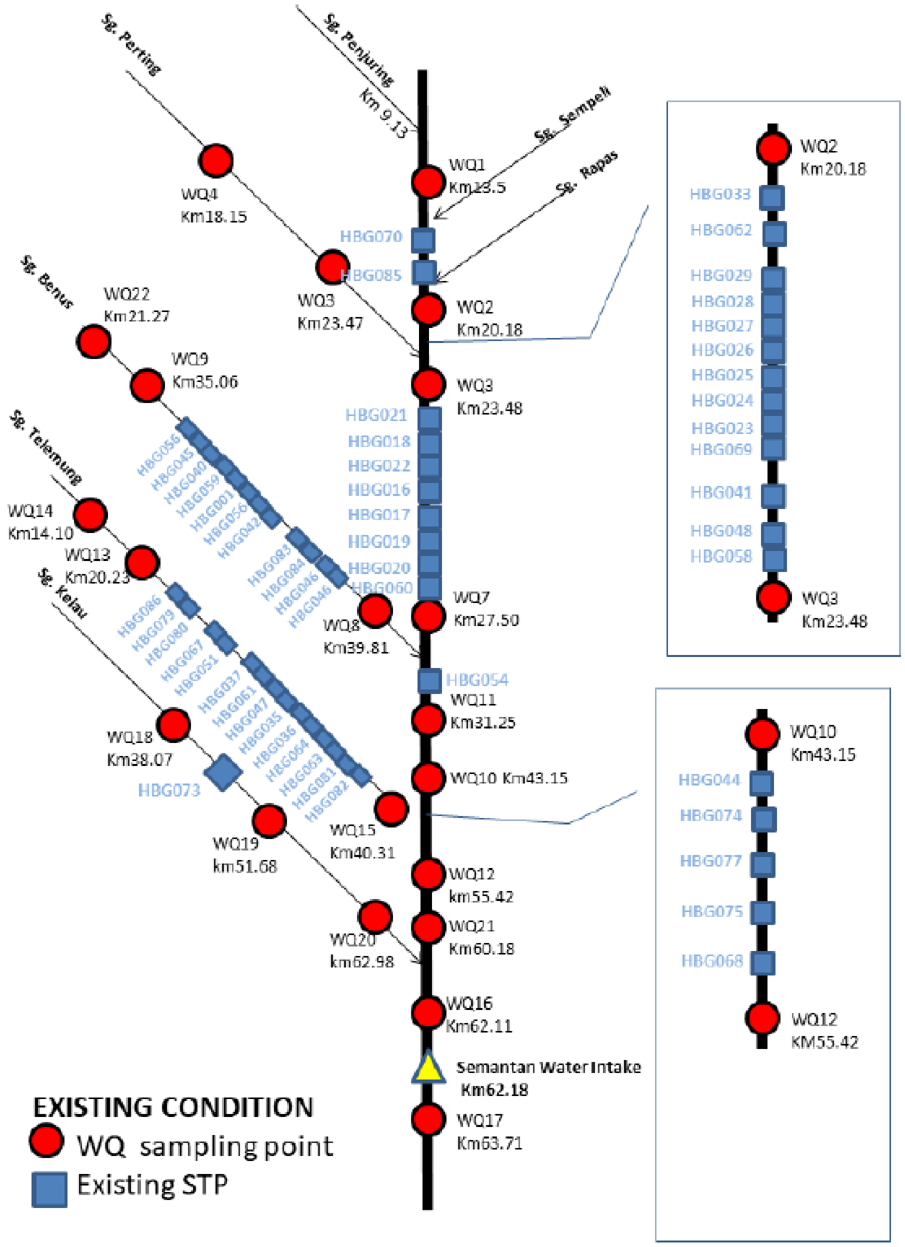

Figure S1. Schematic diagram of Bentong River
